# Respiratory Syncytial Virus Vaccines: Analysis of Pre-Marketing Clinical Trials for Immunogenicity in the Population over 50 Years of Age

**DOI:** 10.3390/vaccines12040353

**Published:** 2024-03-25

**Authors:** Georgios Papazisis, Xanthippi Topalidou, Georgia Gioula, Pablo A. González, Susan M. Bueno, Alexis M. Kalergis

**Affiliations:** 1 Clinical Research Unit, Special Unit for Biomedical Research and Education, School of Medicine, Aristotle University of Thessaloniki, 54124 Thessaloniki, Greece; 2 Department of Clinical Pharmacology, School of Medicine, Aristotle University of Thessaloniki, 54124 Thessaloniki, Greece; xanthippitopalidou@gmail.com; 3 Department of Microbiology, School of Medicine, Aristotle University of Thessaloniki, 54124 Thessaloniki, Greece; 4 Millennium Institute on Immunology and Immunotherapy, Facultad de Ciencias Biológicas, Pontificia Universidad Católica de Chile, Santiago 8320000, Chileakalergis@bio.puc.cl (A.M.K.); 5 Facultad de Medicina, Pontificia Universidad Católica de Chile, Santiago 8320000, Chile

**Keywords:** immunosenescence, inflammaging, vaccinology, respiratory syncytial virus, aging, elderly, clinical trials

## Abstract

Immunosenescence refers to age-related alterations in immune system function affecting both the humoral and cellular arm of immunity. Understanding immunosenescence and its impact on the vaccination of older adults is essential since primary vaccine responses in older individuals can fail to generate complete protection, especially vaccines targeting infections with increased incidence among the elderly, such as the respiratory syncytial virus. Here, we review clinical trials of both candidate and approved vaccines against respiratory syncytial virus (RSV) that include adults aged ≥50 years, with an emphasis on the evaluation of immunogenicity parameters. Currently, there are 10 vaccine candidates and 2 vaccines approved for the prevention of RSV in the older adult population. The number of registered clinical trials for this age group amounts to 42. Our preliminary evaluation of published results and interim analyses of RSV vaccine clinical trials indicates efficacy in older adult participants, demonstrating immunity levels that closely resemble those of younger adult participants.

## 1. Introduction

Aging is extensively researched because of the population’s constantly increasing and anticipated lifespan. According to the World Health Organization (WHO), a two-fold increase in the population over 60 years of age is expected by the year 2050, reaching 2.1 billion people. Interestingly, the increase mentioned above is expected to include a significant proportion of the population in low- and middle-income countries [1]. Significantly, viral and bacterial infections reduce the overall health in this population due to disease resulting from alterations in the immune system following aging [2]. The term “immunosenescence” describes the sequence of immune system adaptations that occur with advanced age [3,4,5]. As a result, the elderly population responds in a downmodulated manner to pathogen exposure as compared with the younger population. At the same time, concomitantly, autoimmunity and the development of malignancies are also associated with immune dysregulation [3]. Older individuals are considered to be in a state of chronic pro-inflammation due to the dysregulation of the immune system and decreased elimination of antigens, scientifically known as “inflammaging” [6,7]. Inflammaging is mediated through cells developing a senescence-associated secretory phenotype (SASP), which triggers low-grade immunity [8,9,10,11]. A multifaceted attribute orchestrates this chronic inflammation process, shaped by environmental and metabolic factors [8]. Noteworthy is the influence of advancing age, which extends to both parts of the immune response, innate and adaptive immunity [11], and immune remodeling leads to the development of systematic diseases, raises the risk of infections, and can differentiate the antibody response to vaccinations [6,12].

### 1.1. Innate Immunity

Toll-like receptors (TLRs) are expressed in phagocytic cells, mediating significant processes related to innate immunity. Dysfunction and altered expression in TLRs appear to be present in the late years of life [8]. By analyzing phagocytic cells, older individuals have been observed to maintain the same number of total monocytes, myeloid dendritic cells (DCs), and neutrophils as younger adults. However, plasmacytoid DCs—which are involved, among other things, in responses to viruses and autoimmune responses—decrease in number [8,13]. Alterations in the functionality of phagocytic cells with imbalanced cytokine release are seemingly a critical factor in the differentiated innate immune response to pathogens [8]. Phagocytosis and the presentation capacity of antigens in DCs also seem weakened [8]. Natural killer (NK) cells are less cytotoxic in the older adult population due to the differentiation of specific subsets and display alterations in cytokine release [14]. Overall, innate immune system dysfunction results in impaired adaptive immune system activation.

### 1.2. Adaptive Immunity

#### 1.2.1. T Cell Immunity

Thymic degeneration and the dysfunctional release of cytokines by thymic cells occurring during aging directly impact the number of new circulating naïve T cells and affect, to a greater extent, CD8^+^ T cells [15]. However, hematopoietic stem cells (HSCs) in the bone marrow remain functional and stable in number with aging [16]. Concomitantly, senescent T cells with an extended duration of life and an impaired ability to replicate an increase in peripheral tissues constrain the immune system’s capacity to respond to new antigens adequately [15]. Some effector T cells, rather than developing into memory cells, transition into either a senescent or exhausted state, which can alter vaccine-induced immunity [17]. It is known that the initiation of immune responses from the T cell antigen receptor (TCR) is modified in CD4^+^ T cells, including the down-regulation of target genes in older human cells and the increased expression of genes encoding pro-inflammatory cytokines [18]. An inversely proportional relationship between Th17 and Treg cells, with Th17 cells increasing during age, suggests a possible explanation for the chronic inflammatory state in elderly individuals [19].

#### 1.2.2. B Cell Immunity

Similar to T cells, naïve B cells experience a numerical decline in the elderly [13]. The B cell repertoire decreases in heterogeneity, especially in people with a reduced general health status [13,20]. Furthermore, the expression of variable B cell receptors (BCRs) is constrained with aging. Additionally, antibodies synthesized from B cells against different antigens show a decreasing trend [8].

Considering these notable alterations in the immune system of the elderly, variations in the responses to vaccination would be anticipated within the older adult population. Nevertheless, progress in understanding the immune alterations related to aging has led to new vaccination approaches to generate higher levels of immunity, as is the case of influenza vaccination, where no lifelong immunity is provided [21,22]. Similarly, new vaccines against Streptococcus, herpes zoster, and COVID-19 effectively induce antibody production and memory immunity in the senior population [17]. Live attenuated vaccines typically ensure lifelong immunity, such as with the yellow fever vaccine the varicella-zoster vaccine, and the measles–mumps vaccine [23]. Nevertheless, instances of disease among vaccinated individuals have been reported for the latter two vaccines during recent outbreaks. It is hypothesized that these occurrences may be attributable to inadequate initial immunization or compromised immunity in certain individuals [23,24]. It is crucial to acknowledge the significant role of vaccination in addressing latent viral infections, exemplified by viruses such as herpes simplex virus, varicella-zoster virus, and human cytomegalovirus [25].

Currently, the Centers for Disease Control and Prevention of the United States (CDC) advises vaccination against COVID-19, influenza, pneumococcal disease, herpes zoster, tetanus, diphtheria, and whooping cough for adults 65 years or older [26]. Additionally, vaccination against respiratory syncytial virus (RSV) was recently approved and suggested for adults aged 60 years or older based on individualized medical evaluation [26]. RSV infection markedly elevates morbidity and mortality rates among the elderly. Lower diagnosis rates of RSV infection have been observed in adults, leading to an undervaluation of the actual disease impact [27,28,29]. Considering the burden of RSV infection in the elderly, several vaccine candidates, some targeting different antigens, have been tested in clinical trials, ensuring multiple approaches in developing protective RSV vaccines for the elderly. Importantly, RSV vaccine research is undergoing significant expansion after the recent market release of two subunit vaccines protecting against RSV-associated lower respiratory tract disease (LRTD) in the elderly population aged 60 years or older, evidencing that vaccines against this pathogen can be achieved after more than six decades of research.

The composition of RSV involves an RNA genome linked to the nucleoprotein (N) forming the RNA-dependent RNA polymerase complex [30]. A large polymerase subunit (L) and a phosphoprotein polymerase cofactor (P) also complete the formation of this complex [31]. The structural integrity of RSV is further reinforced by the matrix (M) protein and the M2-1 protein [30]. These elements are encapsulated within a lipid membrane expressing the fusion (F), attachment (G), and small hydrophobic (SH) proteins [32]. RSV is characterized by genetic variability and classified into two predominant strains, A and B [33]. Strain A seems to be more commonly identified in infected individuals and is concurrently linked to a more pronounced and severe manifestation of the disease [33,34].

In a previous review, we assessed the development of RSV vaccines, encompassing all age target groups and associated outcomes [35]. Here, we present a narrative review of the clinical trials of both candidate and approved vaccines against RSV, which include adults aged ≥50 years, with an emphasis on the evaluation of immunogenicity parameters. This study aims to investigate the immune responses to RSV vaccines within the elderly population, considering the physiological processes of immunosenescence and inflammaging that contribute to altered immune responses.

## 2. Materials and Methods

We conducted searches on the Cochrane Database, the MEDLINE database (PubMed), the ClinicalTrials.gov clinical trial registry, and the WHO International Clinical Trials Registry Platform (ICTRP). Additionally, we extracted data from the European Medicines Agency (EMA), the Centers for Disease Control and Prevention of the United States (CDC), and the U.S. Food and Drug Administration (FDA), as well as from the official websites of pharmaceutical companies forming the RSV vaccine landscape. The search terms for this study included variations of “respiratory syncytial virus”, “vaccine”, “elderly”, and “clinical trial”. The last update of the literature research was on 27 December 2023. A modified PRISMA flow diagram of the research process is presented in the Supplementary Materials (File S1: Modified PRISMA flow diagram). The inclusion criteria comprised clinical trials testing an RSV vaccine candidate or already approved vaccine, enrolling an adult population aged 50 years and above. Clinical trials of candidates in the early stages of development in younger adults were also incorporated into the review under the stipulation that inclusion depended on the company’s intention for market approval as a vaccine for older adults. Variables of interest were the vaccine type and the antigen used, the age group of the participants, and the immunization responses. The exclusion criteria included preclinical phases of development, passive immunization drugs, and clinical trials of the vaccines in different populations. In the following section, a detailed report of the results is presented.

## 3. Results

A summary of the results is presented in Table 1 and Table 2.

### 3.1. mRNA Vaccines

#### 3.1.1. mRNA-1345

mRNA-1345, developed by Moderna, Inc., is under evaluation by regulatory authorities. The company announced in July 2023 the submission of data from clinical trials for vaccine licensure to the European Medicines Agency, Swissmedic, and the Therapeutic Goods Administration in Australia [57]. The results from Phase I clinical trials (NCT04528719, NCT05397223, and NCT05585632) for older adult participants indicated immunity induction in this age group. Specifically, in the NCT04528719 study, the geometric mean fold rises (GMFR) 1 month after vaccination were 12.1–16.6 and 8.7–12.6 for the RSV-A- and RSV-B-neutralizing antibodies, respectively. Reduced GMRF was measured at 6 months and maintained at a minimum level of 4.1. The levels of pre-fusion (PreF)-binding antibody GMFRs followed a similar pattern. These measurements were analogous to the measurements of the younger groups [36]. ConquerRSV (NCT05127434) further tested mRNA-1345 in adults ≥60 years old as a Phase II/III clinical trial. The vaccine efficacy (VE) reached a rate of 83.7% protection against RSV-LRTD, defining the disease with at least two symptoms. Using the definition of three or more symptoms for RSV-LRTD, the VE did not show a substantial difference, with a rate of 82.4%. The study further evaluates the participants’ responses and expects new analyses [57]. Finally, RSVictory (NCT05330975), another Phase III trial, assesses mRNA-1345 in adults ≥50 years old. Moderna recently initiated a Phase III trial (NCT06067230) to test this vaccine in adults 18 years or older with high-risk comorbidities for RSV severe infection. The co-administration of this vaccine with a quadrivalent influenza vaccine for individuals aged 65 years or older is also ongoing as a Phase III clinical study (NCT06060457).

#### 3.1.2. RSV mRNA LNP CL-0059/RSV mRNA LNP CL-0137

Sanofi Pasteur introduced a candidate vaccine for older individuals also using mRNA technology. This vaccine is delivered through one of two lipid nanoparticles (LNPs), namely, LNP CL-0059 or LNP CL-0137. Enrolling a total of 790 participants of two age groups—both younger and older adults—this Phase I/II study (NCT05639894) is anticipated to be completed by 2026.

### 3.2. Subunit/Viral-like Particle (VLP)-Based Vaccines

#### 3.2.1. IVX-A12

The IVX-A12 candidate from Icosavax, Inc., is a virus-like particle (VLP)-based vaccine composed of two partial vaccines, IVX-121 and IVX-241, against RSV and the human metapneumovirus (hMPV), respectively. The F protein in its stabilized prefusion form is the antigen used with the addition of MF59, an oil-in-water emulsion, as the adjuvant. A Phase I clinical trial (NCT05664334) recruited individuals aged 60 to 75 years. The evidence of a prespecified analysis indicates an immunologically feasible combination of the antigens as a single vaccine form. One month after vaccination, there was an approximately sixfold increase in the geometric mean titers (GMTs) of RSV-A-neutralizing antibodies and a threefold increase in RSV-B compared with the titers of placebo recipients. The GMFR reached up to fourfold for RSV-A and threefold for RSV-B with similar results for hMPV antibody titers [38]. After these results, the company announced a Phase II clinical trial (NCT05903183), having already enrolled 264 adults 60 to 85 years of age. Recently, the company shared the interim data from this trial. A substantial elevation, approximately sixfold, in the titers of neutralizing antibodies specific to the RSV-A strain and, concomitantly, a fourfold elevation in the RSV-B strain were reported. The results for hMPV were similarly promising [39].

#### 3.2.2. DPX-RSV(A)

Immunovaccine Technologies, Inc., has developed a subunit vaccine based on the RSV-A small hydrophobic (SH) protein, specifically, the extracellular domain of the protein, representing a new approach that diverges from targeting the F protein as an antigen of interest. In a Phase I first-in-human study (NCT02472548), participants aged 50–64 years of age were stratified into different dose groups. The immunogenicity outcomes for this novel antigen were encouraging. Specifically, vaccines exhibited a tenfold increase in the geometric mean antibody titer ratio at two months. Further, at day 236, they demonstrated nearly a one-hundredfold increase in the GMTR in the participants receiving the vaccine. Immunogenicity was not achieved for the placebo recipients, and they were adjuvanted with an alum formulation. The antibody elevation was observed for up to 180 days following the second dose. High levels of immunogenicity were achieved for this age group of 50–64-year-old participants [40]. The activation of humoral immunity following vaccination reached a level comparable to that observed after natural exposure to the virus [58].

#### 3.2.3. VN-0200

Daiichi Sankyo Co. created VN-0200 against RSV and is currently conducting a Phase II clinical trial (NCT05547087) on adults in Japan in an age range of 60–80 years. The Phase I study (NCT04914520) is already completed, including a wide age range of young and older adults. The antigen of interest is stated as VAGA-9001a, and the adjuvant is MABH-9002b. However, the company did not disclose the specific antigen and adjuvant. Information on the results is not currently published.

#### 3.2.4. BARS13 (ADV110)

BARS13, manufactured by Advaccine Biopharmaceuticals Co., uses the RSV-G protein as the antigen, co-administered with cyclosporine A as the adjuvant [59,60]. Evaluation of the vaccine in younger adults aged 18–45 years in a Phase I clinical trial (NCT04851977) revealed a dose-dependent induction of immunity regarding specific antibodies against the RSV-G protein [59]. Subsequently, a Phase II study (NCT04681833) followed to assess the vaccine in older adults aged 60–80 years. The enrollment was completed with 125 individuals, and the expected completion date is 2024.

#### 3.2.5. DS-Cav1 (VRC-RSVRGP084-00-VP)

DS-Cav1 was developed by the National Institute of Allergy and Infectious Diseases (NIAID) with the aim of inducing immunity against the PreF protein of RSV. Modifications have led to the stable form of the PreF protein [61,62]. The vaccine was successfully tested in a Phase I clinical trial (NCT03049488) in adults 18–50 years of age. Evidence of immunity arose for the participants receiving the unadjuvanted formulation. The dose-dependent production of neutralizing antibodies for both RSV strains was measured up to week 44. Interestingly, mucosal immunity was also activated. One of the vaccine’s target age groups is older adults [41]. Various modifications were subsequently tested to achieve elevated levels of immunity [63,64,65].

#### 3.2.6. Arexvy™

Arexvy™, by GlaxoSmithKline (GSK) plc., is the first vaccine to receive authorization from the FDA for public use. This vaccine is indicated for adults aged 60 years or older and aims to protect this population from lower respiratory tract disease related to RSV infection [66]. Subsequently, the EMA and Medicines and Healthcare Products Regulatory Agency (MHRA) provided market licensure for the vaccine with the same indication [67,68]. The target antigen is the RSV-F protein in the stable prefusion form, delivered with adjuvant system 01 (AS01E) to enhance the immune response. The initial assessment in two Phase I clinical trials (NCT03814590 and NCT04090658) included adults aged 60–80 years old. Higher titers of specific IgG antibodies, neutralizing antibodies, and CD4^+^ T cells were measured after vaccination with peak titers on day 31. Maintenance up to a year after immunization was noted. Cell-mediated immunity activation is an important focus for older adults [42,43]. A Phase II study (NCT04657198) was designed as an extension trial of the previous Phase I trial, NCT03814590, revaccinating the older adult participants of the initial study 18 months after the last vaccination in the parent study. Following the initial trial, the responses endured with a diminished intensity until revaccination. Subsequent to revaccination, a notable increase in specific IgG and neutralizing antibodies, accompanied by the further reactivation of CD4^+^ T cell immunity, was described. These results signify the enhancing impact of a revaccination schedule in older adults [44]. A Phase IIb open-label clinical trial (NCT05921903) is actively enrolling individuals 50 years of age or older. The trial aims to assess responses in patients deemed at high risk for RSV-associated lower respiratory tract disease. A total of nine Phase III clinical trials (NCT04732871, NCT04841577, NCT04886596, NCT05059301, NCT05559476, NCT05568797, NCT05590403, NCT05966090, and NCT05879107) are registered. The results of the NCT04732871 trial up to month 6 were similar to those of the previous studies, with high levels of humoral and cellular immunity, lasting above the baseline titers up to month 6 [45]. The NCT04886596 Phase III clinical trial enrolled 24,966 participants aged 60 years or older. The measured vaccine efficacy in this trial demonstrated an 82.6% efficacy against RSV-associated lower respiratory tract disease, augmenting to 94.1% for severe cases of RSV-associated lower respiratory tract disease. Additionally, an efficacy rate of 71.7% was reported for the outcome of one or more RSV-associated acute respiratory infection episodes. No significant differences were observed between adults 60–69 and 70–79 years and adults with comorbidities. Specifically, the vaccine efficacy was estimated at 93.8% for the age group of 70–79 years, 92.9% for vaccinees with a pre-existing condition of frailty, and 94.6% for participants with comorbidities. The authors highlighted that responses in adults 80 years or older and frail individuals need a cautious examination [47]. The study is presently in progress.

#### 3.2.7. Abrysvo™

Abrysvo™ is also authorized by the FDA and EMA, sharing the same indication as Arexvy™ for adults aged 60 years or older. Pfizer Inc. developed the vaccine as a bivalent subunit candidate encoding the RSV-F protein in the stabilized PreF form from both RSV strains [69,70]. The first-in-human clinical trial (NCT03529773) for Abrysvo™ included 1235 younger and older adult participants. The positive immunogenicity outcomes, characterized by elevated antibody titers against both RSV-A and RSV-B and specific IgG titers, indicated a sustained immunity that, although diminishing, remained above baseline for up to 12 months post-vaccination [49]. These results manifested, to a comparable extent, in younger and older adult participants [48]. A Phase Ib co-administration study (NCT05788237) with another vaccine candidate targeting influenza was recently completed, but the results have not been announced. In the Phase II development stage, studies included non-pregnant and pregnant women (NCT04071158 and NCT04032093), and younger adults were included in an RSV challenge study (NCT04785612). within the framework of this controlled viral exposure protocol, the vaccine exhibited effectiveness regarding the manifestation of clinical symptoms associated with RSV disease [50]. A Phase III clinical trial (NCT04424316) in pregnant women was recently completed. RENOIR (NCT05035212), an active Phase III clinical trial, has enrolled 37,633 adults 60 years and older. A primary analysis of the data up to July 2022 was published. The efficacy was determined to be 66.7% in preventing RSV-related lower respiratory tract illness characterized by the presence of at least two or more symptoms. This efficacy rate increased to 85.7% for cases of illness with at least three or more symptoms. The corresponding rate for acute illness was 62.1%. The vaccine was protective throughout the entire RSV season. Furthermore, specific analyses were conducted across three age groups (60–69 years, 70–79 years, and ≥80 years) and among individuals categorized as high risk for illness. The results were encouraging, revealing no significant differences in the responses across groups. It remains to be clarified whether the responses can be sustained across subsequent RSV seasons in order to determine the vaccination schedule. Additionally, further investigation is required to ascertain the nature of responses in individuals with an immunodeficiency state [51]. In a separate Phase III clinical trial (NCT05096208), three lots of this vaccine underwent testing in younger adults. A Phase III trial (NCT05301322) involving co-administration with a seasonal inactivated influenza vaccine was recently completed. MONET (NCT05842967) is an active Phase III trial that has already enrolled 858 adults with a high-risk condition of developing severe illness. Two sub-studies are planned, including individuals aged at least 60 years.

### 3.3. Live Attenuated/Chimeric Vaccines

#### 3.3.1. BLB-201

Blue Lake Biotechnology’s candidate vaccine BLB-201 is based on the full-length RSV-F protein, is administered through the nasal route, and utilizes the viral live attenuated vector parainfluenza virus type 5 (PIV5) [71,72]. In an early Phase I study (NCT05281263), BLB-201 underwent testing across two age groups, adults aged 18–59 and 60–75. BLB-201 effectively boosted specific serum and mucosal antibody production in both age groups. The mucosal immune response was activated to a greater extent in younger participants, suggesting a potential adjustment in dosage or administration schedules for the elderly. Interestingly, older adult participants showed strong cytotoxic CD8^+^ T cell immunity [54]. A Phase I/II study (NCT05655182) was also initiated for the pediatric population.

#### 3.3.2. rBCG-N-hRSV

Investigators at the Pontificia Universidad Católica de Chile created rBCG-N-hRSV, a recombinant vaccine using a live attenuated *Mycobacterium bovis* bacillus Calmette-Guérin (BCG) platform. The antigen expressed through BCG is the nucleoprotein (N) of RSV [73,74,75]. The incorporation of this combination provides the benefit of a bivalent vaccine [74]. The first-in-human Phase I clinical trial included younger adults up to the age of 50 years. Following vaccination, elevated levels of specific antibodies against both antigens were observed, with a more pronounced effect observed in participants who received the highest dose [55]. Additional analyses regarding immunogenicity aspects were conducted, enhancing the positive data from the Phase I trial in younger adults. Assessment of the results will help further the planning of the upcoming Phase II clinical trials [56].

### 3.4. Recombinant Vector-Based Vaccines

#### 3.4.1. MVA-BN-RSV/Ad26.RSV.preF

The recombinant vector-based vaccine MVA-BN-RSV from Bavarian Nordic uses the modified vaccinia Ankara virus, altered to induce immunity against the F, G, N, and M2 proteins of RSV [76]. Recently, the company announced the withdrawal of the development of this vaccine from its pipeline because one of the primary outcomes of the Phase III clinical trial (NCT05238025) regarding vaccine efficacy was not achieved [77]. Previously, Janssen Pharmaceuticals stopped the further development of Ad26.RSV.preF in Phase III in the context of prioritizing the development of medicines for patients [78]. In Ad26.RSV.preF, Adenovirus 26 acted as the vector encoding the RSV-F protein [79].

#### 3.4.2. RSV/Flu-01E

Following the discontinuation of MVA-BN-RSV and Ad26.RSV.preF, the Research Institute of Influenza in Russia announced a Phase I trial (NCT05970744) for a new recombinant vector-based vaccine candidate for RSV. RSV/Flu-01E uses influenza as the vector to encode the F protein of RSV. Results related to this study are not yet available. The influenza virus has previously undergone testing as a vector-based vaccine delivered intranasally, encoding for the F and G proteins [80]. Encouraging outcomes were observed in preclinical studies [80].

## 4. Discussion

Comprehension of alterations related to the aging of the immune system guides the strategic design of vaccines, aiming to address the challenges of achieving a potent and safe vaccine. Vaccine platform and antigen selection play key roles in developing effective vaccines for specific age populations. Live attenuated RSV vaccine candidates are predominantly designed for the pediatric population, as their efficacy tends to be less pronounced in older adults [81], and the development of recombinant subunit, vector-based, conjugated, or mRNA vaccines is somewhat preferred for older adults [82]. The initiation of a vaccination schedule against a new pathogen in older adults is not indicated using live attenuated vaccines because of a link to a higher rate of adverse events. This observation highlights the significance of the early administration of live attenuated vaccines at younger ages, combined with later booster dosages of vaccines based on inactivated or subunit platforms later in life [83]. The identification of the appropriate antigen or combination of antigens is also important for the success of a vaccination, especially in older adults. Given certain study results, antibodies that specifically recognize the PreF conformation of the F protein exhibit higher efficacy than those targeting the post-F conformation. This observation has led to the selection of the PreF-stabilized protein as an antigenic target for numerous new vaccines under development, optimizing the selection of antigens. It is supposed that mutations in RSV antigenic sites may diminish the efficacy of a vaccine encoding only these specific sites. Thus, including additional antigens, such as the G protein, seems to be a viable option to optimize host immune responses to the virus [84]. The primary point of divergence and determination for each viral strain lies in the RSV-G protein [85,86]. This feature explains the possibility of strain specificity in responses when targeting the G protein. However, a specific central part of the G protein remains unchanged between RSV-A and RSV-B and induces neutralizing activity [86,87].

Regarding the antigen of interest, dose selection is also of great importance for the resulting immunity. A high-dose antigen can significantly boost the titers of specific antibodies. Furthermore, an immunization schedule incorporating repeated vaccinations assists in establishing long-lasting immunity. Different administration routes can also be tested, such as the intradermal route, with the additional advantage of skin antigen-presenting cell function for the presentation of antigens [88]. Adjuvant use in vaccine formulation is another strategy to enhance the host’s immunity against a co-administrated antigen [89]. Aluminum salts and MF59, an oil-in-water emulsion, are commonly added as immunostimulators during vaccine development [88]. It seems that targeted vaccine development for this specific population may overcome the impairment of the aging immune system [17].

An estimation model was designed to assess the potential impact of RSV disease in the United States, considering hypothetical vaccination. According to the estimation, approximately 2.0 million individuals 60 years or older could be protected with vaccination against clinically manifested RSV-associated acute respiratory illness. This corresponds to approximately 50% of the total annual cases estimated for older adults [90]. A separate economic model predicted the prevention of around one-third of the total cases of RSV-associated hospitalizations and deaths in the United States for elderly with an RSV vaccination [91]. According to the records retrieved from the Respiratory Syncytial Virus-Associated Hospitalization Surveillance Network for the period spanning July 2022 to June 2023, more than half of older adults requiring hospitalization due to RSV infection were 75 years of age or older. Among this demographic, clinical comorbidities were prevalent, contributing to an elevated demand for intensive care and interventional ventilation. Furthermore, individuals of younger age from diverse ethnic backgrounds exhibited heightened vulnerability to this disease, suggesting a varied age distribution across affected individuals. The decision for vaccination should be guided, considering these parameters for people aged 60 years or older [92].

GSK and Pfizer’s RSV vaccines are publicly accessible for active immunization in adults aged 60 years or older. Positive outcomes derived from Phase III clinical trials indicate a minimum of moderate to high efficacy rate in protecting against RSV-related LRTD for two RSV seasons. Currently, there is a lack of evidence regarding vaccination’s impact on hospitalizations and mortality. Post-marketing studies should elucidate the safety of vaccines, focusing specifically on adverse neurological events that are observed in preclinical trials [93]. The contribution of the clinical assessment of the patient’s health status is important for the decision to vaccinate against RSV in patients 60 years of age or older, according to the CDC [94]. An off-label immunization could be applied according to German organizations for individuals at high risk for the development of severe RSV disease, such as immunocompromised individuals or those with hematologic malignancies or burdened cardiopulmonary function [95]. A cost-effectiveness analysis in the United States, considering the two approved vaccines, has estimated the impact of vaccination, taking into account a coverage level similar to that seen with influenza vaccination. In this context, a decrease in the burden of the disease was estimated. Nevertheless, the real-world data may exhibit considerable variations, considering the inherent limitations of an economic model [96]. Another similar model also evaluated the impact of two vaccines on older adults in Hong Kong. Vaccination with either one of the two available vaccines leads to the prevention of RSV cases, increasing the quality of life as evaluated in an economic cost-effectiveness study. The vaccine price and the incidence of RSV infections determine the outcomes in real-world data and shape the economic impact of vaccination strategies [97]. mRNA-1345 has shown positive outcomes in preclinical studies, and these findings have already been submitted for evaluation to obtain approval. Currently, three candidates have proceeded to Phase II clinical trials, and six vaccine candidates are in the early stage of development. Most vaccine candidates use the F protein as the target antigen, while various antigens are also being tested.

While undergoing the first RSV season, wherein two vaccines are available for the active immunization of the elderly, epidemiological, safety, and immunological data will contribute to meeting the criteria for the safe and effective prevention of RSV in vulnerable populations. Post-marketing surveillance constitutes an integral component of this process and can assess safety signals that emerge during clinical trials. Targeted Phase III clinical trials have already evaluated the effect of the coadministration of RSV vaccines compared with vaccines targeting other respiratory viruses, as well as responses in specific sub-groups at a heightened risk of severe RSV infection. Vaccination programs will also be shaped based on the duration of the induced immunity and the costs of vaccines, depending on the country. Various vaccines are under development for this vulnerable population, targeting different antigens using different vaccine types. The RSV vaccine landscape is a rapidly changing field, as more vaccine developments are anticipated. The targeted development of vaccines tailored for the elderly, appropriately adjusted to the changes in their immune systems, appears to yield successful outcomes.

## 5. Conclusions

Vaccine development remains challenging for the elderly due to the limited understanding of immunosenescence and its implications for vaccination. Infections such as RSV have direct and indirect impacts on morbidity and mortality within this population. Significant progress has been made in vaccine development in this field, addressing the challenges posed by immunosenescence and inflammaging. with two approved vaccines and a substantial number of clinical trials for additional vaccine candidates underway, a significant optimization of the immunization coverage for this age group is expected, enhancing public health outcomes and the quality of life of the elderly. Current ongoing and additional post-marketing studies are anticipated to identify potential safety signals, contributing to the delivery of safe vaccines to this population.

## Figures and Tables

**Table 1:** Vaccines against respiratory syncytial virus for adults over 50 years old.

Vaccine Technology	Vaccine Candidate	Manufacturer	Target
mRNA	mRNA-1345	Moderna, Inc., Cambridge, MA, USA	F protein
	RSV mRNA LNP CL-0059 or LNP CL-0137	Sanofi Pasteur (a Sanofi Company), Lyon, France	Unknown antigen
Subunit and viral-like-particle-based vaccines	IVX-A12	Icosavax, Inc., Seattle, WA, USA	F protein
	DPX-RSV(A)	Immunovaccine Technologies, Inc., Halifax, NS, Canada	SH protein
	VN-0200 (undefined antigen and adjuvant)	Daiichi Sankyo Co., Tokyo, Japan	VAGA-9001a (unknown antigen)
	BARS13 (ADV110)	Advaccine Biopharmaceuticals Co., Beijing, China	G protein
	DS-Cav1	National Institute of Allergy and Infectious Diseases (NIAID), Bethesda, MD, USA	F protein
	Arexvy™	GlaxoSmithKline plc. (GSK plc.), Brentford, UK	F protein
	Abrysvo™	Pfizer Inc., New York, NY, USA	F protein
Live attenuated/chimeric	BLB-201	Blue Lake Biotechnology, Inc., Athens, GA, USA	F protein
	rBCG-N-hRSV	Pontificia Universidad Católica de Chile, Santiago, Chile	N protein
Recombinant vector-based vaccine	RSV/Flu-01E	Research Institute of Influenza, Saint Petersburg, Russia	F protein

**Table 2:** Respiratory syncytial virus vaccines: clinical trials in adults over 50 years of age.

Vaccine Candidate	Clinical Trial Phase/Population	Outcome
mRNA-1345	NCT04528719 (I) ≥18 to ≤49 years ≥65 to <80 years	Geometric mean fold rise (GMFR) one-month post-vaccination for older adult participants: 12.1–16.6 for RSV-A and 8.7–12.6 for RSV-B-neutralizing antibodies (nAbs). Similar levels for PreF-binding antibodies. Results analogous to those of the younger adults [36].
	NCT05397223 (I) 18–75 years	Active trial. Tests mRNA vaccines against SARS-CoV-2, seasonal influenza, respiratory syncytial virus (RSV), and cytomegalovirus.
	NCT05585632 (I) 50–75 years	Active trial. Tests multi-component mRNA vaccines against influenza, RSV, and SARS-CoV-2.
	ConquerRSV NCT05127434 (II/III) ≥60 years	Vaccine efficacy (VE) against RSV-low respiratory tract disease (LRTD). Manifested with ≥2 clinical symptoms: 83.7%. Manifested with ≥3 clinical symptoms: 82.4%. New analyses from the study are expected [37].
	RSVictory NCT05330975 (III) ≥50 years	Active trial. Co-administration with seasonal influenza vaccine or SARS-CoV-2 vaccine.
	NCT06067230 (III) ≥18 years with high-risk comorbidities	Currently enrolling participants.
	NCT06060457 (III) ≥65 years	Currently enrolling participants. Co-administration with high-dose quadrivalent seasonal influenza vaccine.
RSV mRNA LNP CL-0059 or LNP CL-0137	NCT05639894 (I/II) 18–50 years and ≥60 years	Active trial.
IVX-A12 (RSV and hMPV)	NCT05664334 (I) 60–75 years	Geometric mean titers (GMTs) one-month post-vaccination: sixfold increase in RSV-A and threefold increase in RSV-B nAbs. GMFR: 4-fold for RSV-A and 3-fold for RSV-B. Analogous results for human metapneumovirus (hMPV) titers [38].
	NCT05903183 (II) 60–85 years	Interim data: GMTs: approximately sixfold elevation in RSV-A nAbs and fourfold elevation in RSV-B nAbs [39].
DPX-RSV(A)	NCT02472548 (I) 50–64 years	Encouraging immunogenicity outcomes for this novel antigen (RSV-A SHe). GMTR. Two months post-vaccination: 10-fold increase. Day 236: a nearly 100-fold increase. The alum formulation did not exhibit these immunogenicity results. Duration of up to 180 days after the second vaccine dose. Humoral immunity activation comparable to that after natural exposure [40].
VN-0200 (undefined antigen and adjuvant)	NCT04914520 (I) ≥20 and ≤50 years ≥65 and ≤80 years (Japan)	Completed trial with no published results.
	NCT05547087 (II) 60–80 years (Japan)	Active trial.
BARS13 (ADV110)	NCT04851977 (I) 18–45 years	Dose-dependent induction of RSV-G-specific antibodies [40].
	NCT04681833 (II) 60–80 years	Active trial.
DS-Cav1	NCT03049488 (I) 18–50 years	Dose-dependent production of nAbs against both strains up to week 44, including induction of mucosal immunity. Modifications are being investigated [41].
Arexvy™	NCT03814590 (I/II) 18–40 years 60–80 years	Dose and adjuvant selection. The high-dose formulation induced analogous increases in specific nAb titers in older adults with the maximum effect after the first vaccination. Elevation of geometric mean frequencies (GMFs) of CD4+ T cells. Duration: a year post-vaccination [42].
	NCT04090658 (I) 60–80 years (Japan)	Titers of specific IgGs and nAbs against both strains of RSV underwent alterations similar to those observed in a previous study [43].
	NCT04657198 (II) ≥60 years participating in the NCT03814590 trial	Specific IgGs and nAbs notably increased after revaccination. Further reactivation of CD4+ T-cell immunity. Results supporting a revaccination schedule in older adults [44].
	NCT05921903 (IIb) ≥50 years at a high risk for RSV-LRTD	Currently enrolling participants.
	NCT04732871 (III) ≥60 years	Results up to month 6. High measured levels of humoral and cellular immunity, lasting above baseline [45].
	NCT04841577 (III) ≥60 years	Co-administration with seasonal quadrivalent influenza vaccine. It was demonstrated that the co-administration is as equally effective as the separate administration of the two vaccines with a one-month interval [46].
	NCT04886596 (III) ≥60 years	VE against RSV-LRTD: 82.6%. VE against severe RSV-LRTD: 94.1%. VE against ≥1 case of RSV-acute respiratory infection (ARI): 71.7%. VE in the age group of 70–79 years: 93.8%. VE for vaccines with pre-existing frailty: 92.9%. VE for participants with comorbidities: 94.6%. The study remains in progress [47].
	NCT05059301 (III) ≥60 years	Study of 3 different lots of the vaccine. Immunity was elicited at comparable levels across different lots.
	NCT05559476 (III) ≥65 years	Co-administration with high-dose quadrivalent influenza vaccine. Study completed without published results.
	NCT05568797 (III) ≥65 years	Co-administration with an adjuvanted inactivated influenza vaccine. Study completed without published results.
	NCT05590403 (III) 50–59 years at high risk for RSV disease and ≥60 years	Active trial.
	NCT05966090 (III) ≥50 years	Active trial. Co-administration trial with herpes zoster recombinant subunit vaccine.
	NCT05879107 (III) ≥60 years	Active trial. Co-administration trial with a 20-valent pneumococcal conjugate vaccine (PCV20).
Abrysvo™	NCT03529773 (I/II) 18–85 years	Elevated Ab titers against both RSV strains and specific IgG titers, overcoming baseline up to 12 months. Similar distribution of results between the two groups [48,49].
	NCT05788237 (Ib) ≥50 years	Co-administration with influenza vaccine. Study completed with no released results.
	NCT04785612 (II) 18–50 years	RSV challenge trial. Vaccine effective against manifestation of clinical symptoms associated with RSV disease [50].
	NCT05886777 (II) ≥65 years	Active trial. Tests vaccination against COVID-19, RSV, and influenza as either combined or separate vaccinations.
	RENOIR/NCT05035212 (III) ≥60 years	Interim data. VE: 66.7% in preventing RSV-related lower respiratory tract infection (LRTI) with ≥2 symptoms and 85.7% for cases of illness with ≥3 symptoms. VE against RSV-ARI: 62.1%. Duration: entire RSV season. No differences in specific analyses between individuals 60–69 years, 70–79 years, ≥80 years, and those at high risk for illness [51].
	NCT05096208 (III) 18–49 years	Tested 3 different lots of the vaccine formulation and demonstrated comparability of the immune responses [52].
	NCT05301322 (III) ≥65 years	Co-administration with seasonal inactivated influenza vaccine. Immunity induction was non-inferior after the co-administration of the two vaccines compared with separate administration [53].
	MONET/ NCT05842967 (III) ≥18 years at high risk for severe illness	Active trial.
	NCT06077968 Population	Retrospective trial based on real-world healthcare data.
Arexvy + Abrysvo (University of Rochester)	NCT06077149 (IV) ≥60 years	Recently posted. Tests the immune induction between older adults residing in long-term care facilities and the community.
BLB-201	NCT05281263 (I) 18–59 and 60–75 years	Boosted specific serum and mucosal antibody production in both groups, with mucosal immunity activation to a greater extent in younger adults (suggestion of dose/administration adjustment for the elderly). Strong elicitation of cytotoxic CD8^+^ T cell immunity in older adults [54].
rBCG-N-hRSV	EVA-VRS01/ NCT03213405 (I) 18–50 years	Bivalent vaccine. Elevated levels of Abs against both antigens proportional to the dose increase. Upcoming Phase II trial is planned [55,56].
RSV/Flu-01E	NCT05970744 (I) 18–59 and ≥60 years	Active trial.

## Data Availability

Data sharing is not applicable.

## Supplementary Materials

The following supporting information can be downloaded at https://www.mdpi.com/article/10.3390/vaccines12040353/s1, File S1: Modified PRISMA 2020 flow diagram. Reference [98] is cited in the Supplementary Materials.
