# Supplementary material for: Respiratory Syncytial Virus Vaccines: Analysis of Pre-Marketing Clinical Trials for Immunogenicity in the Population over 50 Years of Age
Source: Vaccines (Basel). 2024 Mar 25;12(4):353. doi: 10.3390/vaccines12040353 (PMC11054105; doi:10.3390/vaccines12040353)
Supplement: Supplementary file 1 [file vaccines-12-00353-s001.zip › vaccines-2895799-supplementary.pdf]

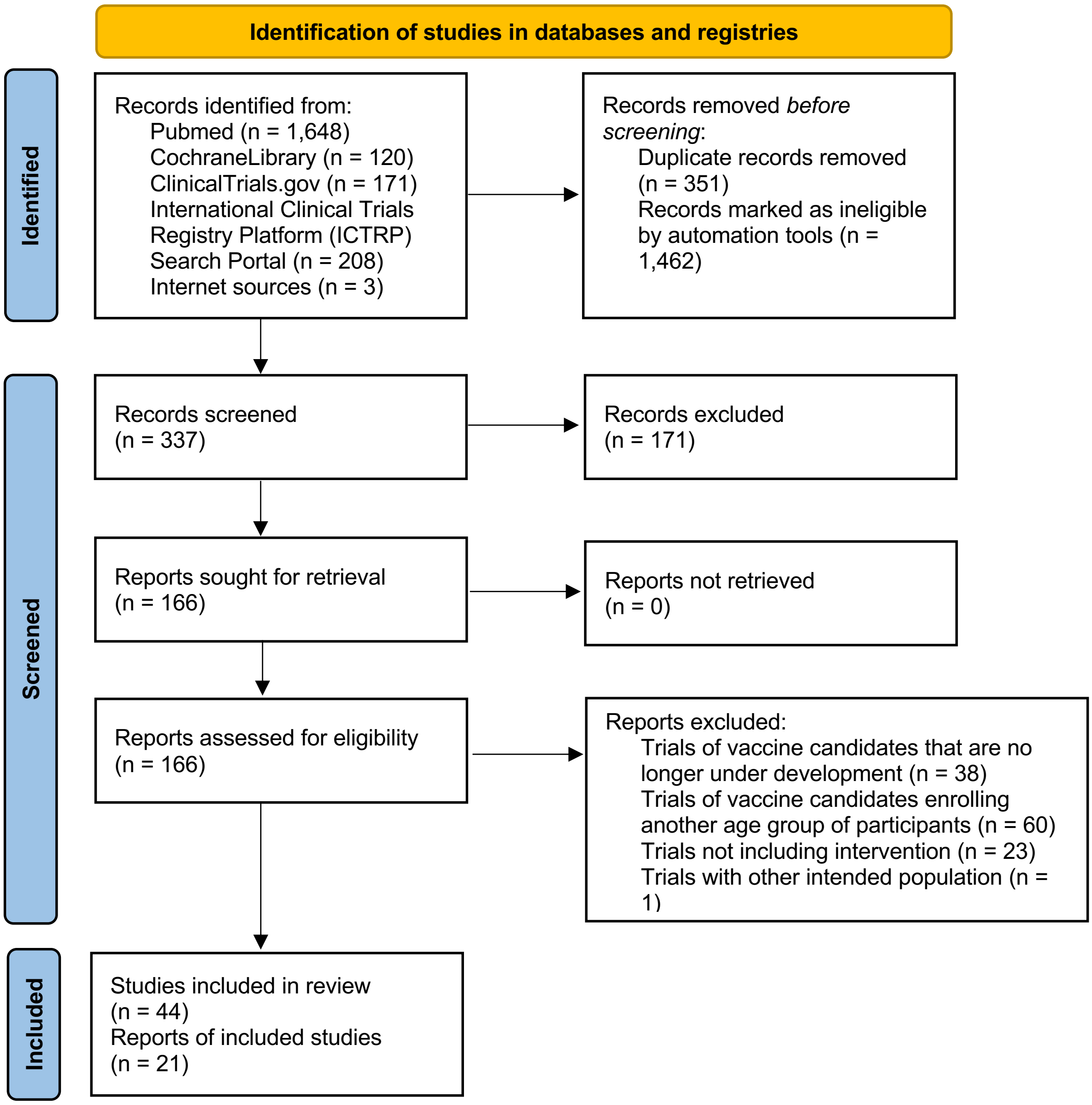

File S1. Modified PRISMA 2020 flow diagram.

From: Page MJ, McKenzie JE, Bossuyt PM, Boutron I, Hoffmann TC, Mulrow CD, et al. The PRISMA 2020 statement: an updated guideline for reporting systematic reviews. BMJ 2021;372:n71. doi: 10.1136/bmj.n71

For more information, visit: http://www.prisma-statement.org/
